# Intermittent Fasting Remodels Mitochondrial Morphology and Bioenergetic Capacity in Brown Adipose Tissue of Obese Mice

**DOI:** 10.1111/apha.70337

**Published:** 2026-11-08

**Authors:** Fernanda Silva Carneiro, Larissa Moreira Dias, Scylas José de Andrade Junior, Matheus Biscaro Rocha, Mariana Lima Ribeiro, Ana Paula Morelli, Gabriel Calheiros Antunes, Adelino Sanchez Ramos Da Silva, Dennys Esper Cintra, José Rodrigo Pauli, Eduardo Rochete Ropelle

**Affiliations:** ^1^ Laboratory of Molecular Biology of Exercise (LaBMEx), School of Applied Sciences University of Campinas Limeira Sao Paulo Brazil; ^2^ Laboratory of Cell Signaling (LabSinCel), Faculty of Medical Sciences University of Campinas Campinas Brazil; ^3^ Postgraduate Program in Rehabilitation and Functional Performance, Ribeirão Preto Medical School, and School of Physical Education and Sport of Ribeirão Preto University of São Paulo (USP) Ribeirão Preto São Paulo Brazil; ^4^ Laboratory of Nutritional Genomics, School of Applied Sciences University of Campinas (UNICAMP) Limeira Sao Paulo Brazil; ^5^ Obesity and Comorbidities Research Center (OCRC), Faculty of Medical Sciences University of Campinas Campinas Brazil; ^6^ National Institute of Science and Technology for Obesity and Diabetes Campinas Brazil

**Keywords:** brown adipose tissue, intermittent fasting, mitochondrial dynamics, mitochondrial metabolism

## Abstract

Brown adipose tissue (BAT) is a specialized adipose tissue that is associated with thermogenesis, endocrine function, and energy expenditure, characterized by a high mitochondrial density. However, obesity induces profound structural and functional remodeling of mitochondria in BAT, compromising their thermogenic and oxidative capacities. On the other hand, intermittent fasting is a non‐pharmacological approach that can enhance mitochondrial function, thereby contributing to weight loss. Despite this, the effects of intermittent fasting on the morphofunctional aspects of BAT mitochondria are not yet well understood. Thus, we investigated the effects of a chronic 16:8 daily intermittent fasting protocol over 8 weeks on the BAT mitochondria of obese mice. Diet‐induced obesity reduced energy expenditure, increased body weight gain, and adiposity within the BAT. Obesity altered BAT mitochondrial morphology, resulting in smaller, more rounded organelles with disrupted cristae. Conversely, intermittent fasting increased energy expenditure, reduced body weight gain and adiposity within the BAT, increased the UCP1 protein content, and restored browning in the BAT. Remarkably, intermittent fasting promoted extensive remodeling of mitochondrial dynamics protein markers and structural profile in BAT, increasing mitochondrial elongation, total cristae length, and cristae alignment. In addition, intermittent fasting increased the maximum intrinsic capacity of respiratory mitochondrial complexes and the content of proteins related to oxidative phosphorylation. These findings demonstrate that intermittent fasting is a potential strategy for regulating mitochondrial dysfunction in BAT during metabolic disorders.

## Practitioner Points

- Diet‐induced obesity increases BAT whitening, leading to circular, and fragmented mitochondria.
- Intermittent fasting increases the maximum intrinsic capacity of respiratory mitochondrial complexes and UCP1 protein content in BAT.
- Intermittent fasting remodels mitochondrial dynamics protein and structure in BAT.

## 1 Introduction

Obesity is a metabolic multifactorial disease characterized by excessive adiposity resulting from a chronic imbalance between energy consumption and expenditure, leading to several changes in the functionality of tissues and organs [1]. Currently, it is considered a global public health problem, since the excessive accumulation of adiposity leads to clinical metabolic dysfunction, promoting associated comorbidities such as Type 2 diabetes, heart diseases, stroke, and different types of cancers [2, 3], beyond tissue‐specific metabolic and physiological changes.

Brown adipose tissue (BAT) is a metabolically active tissue specialized in dissipating energy as heat in a non‐shivering thermogenesis regulatory mechanism, through a process mainly mediated by uncoupling protein 1 (UCP1) due to its abundant mitochondria, orchestrating multiple cellular functions owing to the tissue's high mitochondrial density, dynamic remodeling capacity, and sensitivity to metabolic stress, which is strictly related to energy expenditure impaired by obesity, excessive adiposity, and a chronic low‐inflammation state [4]. Disruption of these processes may compromise systemic energy expenditure, promoting inflammatory signaling, and impairing ATP homeostasis [5, 6, 7, 8]. During obesity, BAT shows characteristics similar to white adipocytes, triggering mitochondrial adaptations and increasing lipid content, leading to a process called “whitening” [8, 9].

Intermittent fasting (IF) is a nutritional non‐pharmacological strategy characterized by alternating periods of partial or complete caloric restriction that emerged as a strong therapeutic approach for its potential to regulate appetite and reduce caloric intake [10, 11]. Among the various protocols, the 16:8 intermittent fasting regimen (16 h of fasting during the inactive cycle followed by an 8‐h feeding during the active cycle) has demonstrated efficacy in enhancing thermogenesis and mitigating excessive weight gain in murine models and humans [12, 13, 14, 15]. In white adipose tissue, IF enhances mitochondrial bioenergetics by stimulating mitochondrial fusion and restoring ultrastructural alterations [16, 17]. IF also upregulates genes involved in lipid oxidation and fatty acid metabolism [18], promotes adipose tissue browning, and promotes the regulation of mitochondrial quality control mechanisms [16]. Studies have shown that IF enhances succinate metabolism to support thermogenic regulation and reduces BAT mass [19]. In addition, it has been demonstrated that intermittent fasting increases the content of thermogenic markers, including UCP1 and PGC‐1α, while decreasing lipid accumulation [20, 21].

The morphofunctional properties of mitochondria are highly sensitive to shifts in the nutritional and metabolic status, with a characteristic mitochondrial ultrastructure, including densely and highly organized cristae and an elongated mitochondrial network [8]. In pathological conditions such as obesity, as well as in response to dietary interventions such as IF, these alterations can significantly impact mitochondrial metabolism [22], with disrupted mitochondrial dynamics in adipose tissues [23], accompanied by alterations in NAD metabolism [24] and activation of mitochondrial stress responses driven by increased reactive oxygen species (ROS) production [25]. These disturbances impair glucose and fatty acid metabolism [26] and contribute to the regulation of whitening and browning processes in both brown and white adipose tissues [27]. In BAT, IF can improve parameters in healthy metabolism [28] and during obesity [29]; however, the effects of chronic IF on mitochondrial structure and function in BAT during obesity remain poorly explored. In this context, the present study investigated the effects of a chronic IF protocol on long‐term diet‐induced obese mice in BAT mitochondrial structure and metabolism, where we expect to observe beneficial effects of intermittent fasting on mitochondrial metabolism, possibly showing a stronger respiratory capacity and improvement in mitochondrial structure.

## 2 Methods

### 2.1 Animals

C57BL/6J male mice were acquired from Multidisciplinary Center for Biological Research (CEMIB) at the University of Campinas (UNICAMP), under the Ethical Committee approval (protocol number CEUA 6005‐1/2022). The animals were maintained under controlled environmental conditions and housed three per cage, at controlled 45%–55% humidity, 22°C–24°C temperature, and 12/12 h light cycles, following the National Council for Animal Control and Experimentation (CONCEA) guidelines. The number of animals and samples used in each experiment is specified in the figure legends.

### 2.2 Experimental Protocol

At the age of 8 weeks, mice were randomly assigned to a standard chow diet (CT) or a 35% high‐fat diet (HFD) formulated in accordance with the AIN‐93G recommendations [30], for 8 weeks. For the subsequent 8 weeks, half of the mice fed a high‐fat diet were subjected to a daily chronic intermittent fasting (IFC) protocol consisting of a 16:8‐h fasting/feeding cycle during the inactive phase (HFD+IFC group). The fasting period began at 6:00 a.m. and finished at 10:00 p.m., where mice were transferred to clean cages during fasting and feeding periods to prevent food hoarding. The remaining high‐fat diet–fed mice (HFD group) and chow‐fed controls were maintained under *ad libitum* feeding conditions for the duration of the protocol. Body weight and food consumption were measured weekly in all groups. At the end of the experiments, both groups were submitted to a 4 h fasting, followed by intraperitoneal anesthesia at 8 a.m. with ketamine (200 mg kg^−1^) and xylazine (20 mg kg^−1^) solution, and cervical dislocation after the absence of reflexes for euthanasia. Adipose tissues were collected after extraction and weighed, and the combined weight of all adipose depots was used as total fat mass.

### 2.3 Comprehensive Lab Animal Monitoring System (CLAMS) Analysis

To evaluate the effects of intermittent fasting on VO_2_ consumption, VCO_2_ production, energy expenditure (EE), and respiratory exchange ratio (RER), 1 week before euthanasia, mice were submitted to the Comprehensive Lab Animal Monitoring System Analysis (CLAMS‐Oxylet M3 system PanLab; Biology Institute, UNICAMP). Initially, mice were individually housed for 48 h, with the final 24 h used to assess oxygen uptake (VO_2_). The respiratory exchange ratio was calculated as the ratio of VO_2_ production to VO_2_ oxygen consumption. For energy expenditure analysis, it was calculated on an hourly basis from VO_2_ and carbon dioxide production (VCO_2_) using the weir equation EE (kcal·min^−1^) = (3.941 × VO_2_) + (1.106 × VCO_2_), adjusted for mice body weight and expressed as Kcal/h, maintaining the same experimental conditions except the fasting protocol during the 48 h of analysis.

### 2.4 Glucose Tolerance Test

The glucose tolerance test (GTT) was performed after 12 h of fasting at 9 a.m. For the analysis, glucose levels were measured (basal levels) and followed by an intraperitoneal injection of glucose (2 g/kg of body weight), and the blood glucose levels were measured from the tail vein at 0 (basal levels), 15, 30, 60, 90, and 120 min after glucose administration using a glucose meter (Accu‐Chek, Germany).

### 2.5 Infrared Thermography Analysis

To assess in vivo brown adipose tissue (BAT) activation and thermogenic activity, infrared thermography was performed on the interscapular dorsal region of mice. Measurements were conducted using a high‐resolution infrared camera (FLIR Systems, USA), previously calibrated for biological tissues. To minimize external factors, all imaging sessions were performed in a climate‐controlled room kept at a stable ambient temperature (22°C), with acclimatation to the testing room for 30 min. During the procedure, the infrared camera was positioned perpendicularly on a tripod at a standardized distance of 30 cm above the dorsal of each animal. Thermographic images were captured, and the digital thermograms were analyzed using FLIR Tools software (FLIR Systems, USA). The region of interest was defined as a circular area of equal size within the interscapular dorsal zone directly overlying the BAT. The average, maximum, and minimum skin surface temperatures were quantified, and the mean was used to calculate the individual temperature for each animal in the experimental group.

### 2.6 Hematoxylin Eosin (H&E) Staining

For H&E staining, slides with BAT fixed in 4% paraformaldehyde (PFA) for 24 h were first deparaffinized in xylene (2 × 5 min), rehydrated through a descending ethanol series (100%, 95%, 70%, for 30 min each), and rinsed in distilled water. Then, the slides were stained in Harris Hematoxylin for 5 min and differentiated in 1% acid alcohol (1% HCl in 70% ethanol) for a few seconds. After bluing in running water for 5 min, slides were counterstained with Eosin for 20 s. After staining, the slides were dehydrated in ascending ethanol concentrations, cleared in xylene (2 × 5 min), and mounted with coverslips using a synthetic mounting medium. Stained sections were visualized and imaged under a bright‐field microscope and quantified by ImageJ.

### 2.7 Lipid Droplet Quantification and Analysis

Histological images from BAT acquired from H&E staining were used to analyze lipid droplet parameters using ImageJ (National Institutes of Health, USA). For each animal, multiple non‐overlapping fields were manually segmented based on their unstained intracellular vacuolar appearance in H&E sections using the *Freehand Selection* tool. Individual lipid droplet area (μm^2^) was measured using the *Measure* function after setting the appropriate scale for each image. Data from all droplets within each field were pooled to calculate the mean lipid droplet area for each animal. To evaluate BAT remodeling, lipid droplets were classified into area intervals (0–30, 30–60, 60–90, 90–120, and > 120 μm^2^). The number of droplets within each interval was expressed as a percentage of the total lipid droplets quantified for each animal, normalizing the data for differences in droplet number among samples. The percentage distribution of lipid droplets across the predefined size classes was subsequently compared among the experimental groups.

### 2.8 Transmission Electron Microscopy (TEM)

At the end of the experiment, mice were perfused with 8% saline solution followed by Karnovsky's fixative solution (8% paraformaldehyde, 25% glutaraldehyde, and 0.2 M pH 7.3 phosphate buffer), with 2 mm^3^ BAT tissue fragments immersed in Karnovsky's solution at room temperature for 3 h, followed by a 4°C refrigeration until analysis. TEM analysis was performed at the Electron Microscopy Center (CME) of Sao Paulo State University (UNESP, Botucatu, Sao Paulo, Brazil), where the tissues were post‐fixated, washed, dehydrated in ethanol solutions, and sectioned in ultrathin sections with uranyl solution, with the images examined under a Transmission Electron Microscope (Tecnai Spirit, FEI Company).

Images were acquired in randomly selected regions, performed with *n* = 6 mice per group under magnifications of 1050×, 4200×, and 11 500×, depending on the parameter quantified (Supplementary File 1). One sample from the HFD+IFC group was excluded due to technical issues during tissue sectioning, without compromising the statistical power of the analysis. The images were analyzed using the software FIJI (version 2.14.0) for segmentation and the plug‐in Skeletonized (2D/3D) with manual refinement for semi‐automated mitochondrial and cristae quantifications (Supplementary File 2). Each experimental group was calculated to have approximately 200 mitochondria for morphological analysis and 50 mitochondria, resulting in 350–800 individual cristae analyzed per group for detailed cristae analysis. The parameters analyzed were mitochondrial area [31, 32], aspect ratio (ratio between major and minor axes) [31], form factor (to evaluate shape complexity) [32], circularity ratio [33], Feret's diameter (distance between *X* and *Y* axes) [34], Elongation Index [32, 33], intercristae area, total cristae number, cristae number per mitochondrial area [35, 36], total cristae length [37, 38], Angular Alignment Index (index of cristae degrees of alignment relative to the longitudinal axis) [39], and cristae circular deviation (variability between cristae orientation angles, indicating heterogeneity of cristae organization) [40], as well the respective percentage of distribution of each analysis. The detailed methodology for each analysis is available in the Supplementary File 1.

### 2.9 Protein Quantification by Western Blotting (WB)

Frozen BAT tissue fragments were homogenized using ice‐cold RIPA protein extraction buffer with protease (Code A32965, Thermo Fisher, USA) and phosphatase inhibitors (Code A32957, Thermo Fisher, USA). The protein content was quantified using the colorimetric method Bicinchoninic Acid Assay (Pierce BCA Protein Assay, Thermo Fisher, USA). Then, samples were prepared in Laemmli buffer with 15 μg of protein per sample loaded onto SDS‐PAGE gels. Proteins were transferred to 0.45 μm nitrocellulose membranes, and transfer efficiency was verified by Ponceau staining. Membranes were blocked with 5% non‐fat milk for 1 h, incubated overnight with primary antibodies, and subsequently incubated with the corresponding secondary antibodies, with TBS‐T washes performed between incubations. All antibodies are listed in Supplementary Table 1. We used Ponceau as the endogenous control of each analysis to avoid potential modulations by the interventions [41, 42, 43]. The protein bands were visualized and analyzed using the Chemiluminescence ECL Detection Kit (code 6883S, Cell Signaling). The protein content was quantified using the Software ImageJ (NIH, Bethesda, USA). The mitonuclear protein imbalance was evaluated by the protein expression ratio of the mitochondrial protein subunit MTCO1 to the nuclear‐encoded subunit ATP5A, determined by western blotting analysis (Supplementary Table 1).

### 2.10 Gene Expression Quantification by RT‐qPCR

Total RNA was extracted using TRIzol reagent (Invitrogen, Thermo Fisher Scientific, USA), followed by RNA quantification using NanoDrop Spectrophotometer (Thermofisher Scientific, USA). The reverse transcription of RNA into cDNA was performed using iScript cDNA Synthesis Kit (Bio‐Rad, USA) with 1 μg of total RNA. The cDNA content was used to perform RT‐qPCR with iTaq SYBR Green Supermix Kit (Bio‐Rad, USA), analyzed with StepOne Software (Thermofisher Scientific, USA), and quantified using the mean of triplicates by the relative expression calculated using 2−^ΔΔCT^ (Supplementary Table 2).

### 2.11 High‐Resolution Respirometry Analysis on BAT

High‐resolution respirometry analysis was conducted on −80°C frozen tissue samples to assess individual mitochondrial complexes activity using Oxygraph‐2k (Oroboros Instruments, Austria) [44, 45, 46]. For the analysis, total samples were defrosted in 300 μL of 1× MAS buffer (70 mM sucrose, 5 mM KH2PO4, 220 mM mannitol, 5 mM MgCl2, 1 mM EGTA, 2 mM HEPES, pH 7.4) and homogenized on ice using a pestle. Afterwards, the homogenate was centrifuged at 1000×*g* for 10 min at 4°C, and the supernatant was collected, followed by a protein quantification using the Pierce BCA Protein Assay (Thermo Fisher, USA), using a total of 150 μg of protein supernatant per sample. For the respirometry analysis for individual complexes, the tissues were treated with NADH for complex I activity (1 mM), succinate and rotenone for complex II activity (5 mM and 2 μM, respectively), octanoylcarnitine to evaluate dysfunction in the fatty acid oxidation (FAO) pathway (0.1 mM), cyanide‐4‐(trifluoromethoxy)phenylhydrazone (FCCP) titration to evaluate maximum respiratory capacity (0.5 μM), and antimycin A for non‐mitochondrial respiration (2.5 μM).

### 2.12 Lipid Peroxidation Analysis

To evaluate lipid peroxidation and oxidative stress in BAT, thiobarbituric acid reactive substances (TBARS) were quantified using a commercial colorimetric assay kit (Elabscience, China) according to the manufacturer's instructions. For the analysis, BAT samples (20 mg) were washed in cold PBS (0.01 M, pH 7.4) and homogenized in 180 μL of cold PBS using a pistil homogenizer at 4°C. The homogenates were centrifuged (10.000*g*, 10 min, 4°C) to remove insoluble material, and the supernatants were collected and kept on ice for further analysis. Total protein concentration in the supernatant was determined using a Pierce BCA Protein Assay (Thermo Fisher, USA) to normalize the TBARS values. For the colorimetric reaction, 0.1 mL of standard solutions or tissue supernatants was added to glass tubes. Subsequently, 0.1 mL of clarificant and 4 mL of freshly prepared chromogenic agent (a mixture of acid application solution and TBA application solution) were added to each tube and mixed gently. The tubes were sealed with a small puncture and incubated in a water bath at 100°C for 1 h. After cooling to room temperature under running water, the mixture was centrifuged (1.600*g*, 10 min, room temperature), and 0.25 mL of the sediment‐free supernatant was transferred to a 96‐well microplate. Afterward, the absorbance was measured at 532 nm using a microplate reader. TBARS content was calculated using the standard curve and normalized to the protein concentration of each sample.

### 2.13 Statistical Analysis

All the data presented were subjected to a normalization test, with one‐way ANOVA, two‐way ANOVA, or nested one‐way ANOVA with Tukey *post hoc* applied according to the requirements of the analysis. For data presentation, the statistical significance adopted was presented as mean ± standard deviation (SD) and a *p* value of < 0.05. The statistical analysis was performed in GraphPad Prism (version 8.0.2). To evaluate EE without the confounding effect of body mass, a standard analysis of covariance (ANCOVA) Type III was performed, with pairwise comparisons of the estimated marginal means (EMMeans) using Tukey's post hoc adjustment. Statistical analyses for ANCOVA were performed in R (version 4.4.0) using *car* and *emmeans* packages, with significance set as *p* < 0.05.

## 3 Results

### 3.1 Intermittent Fasting Attenuates Obesity‐Induced Body Weight Gain and Adipose Tissue Accumulation

As expected, after 8 weeks of high‐fat diet (HFD), mice showed an increased body weight compared to the control group. However, in the next 8 weeks, chronic intermittent fasting (IFC) prevented body weight gain compared with the HFD group (Figure 1A,B), without altering food intake, except during the 6th week of the protocol (Figure 1C). In the last week of the experiment, we observed that intermittent fasting completely prevented the mice from developing glucose intolerance (Figure 1D).

**FIGURE 1:**
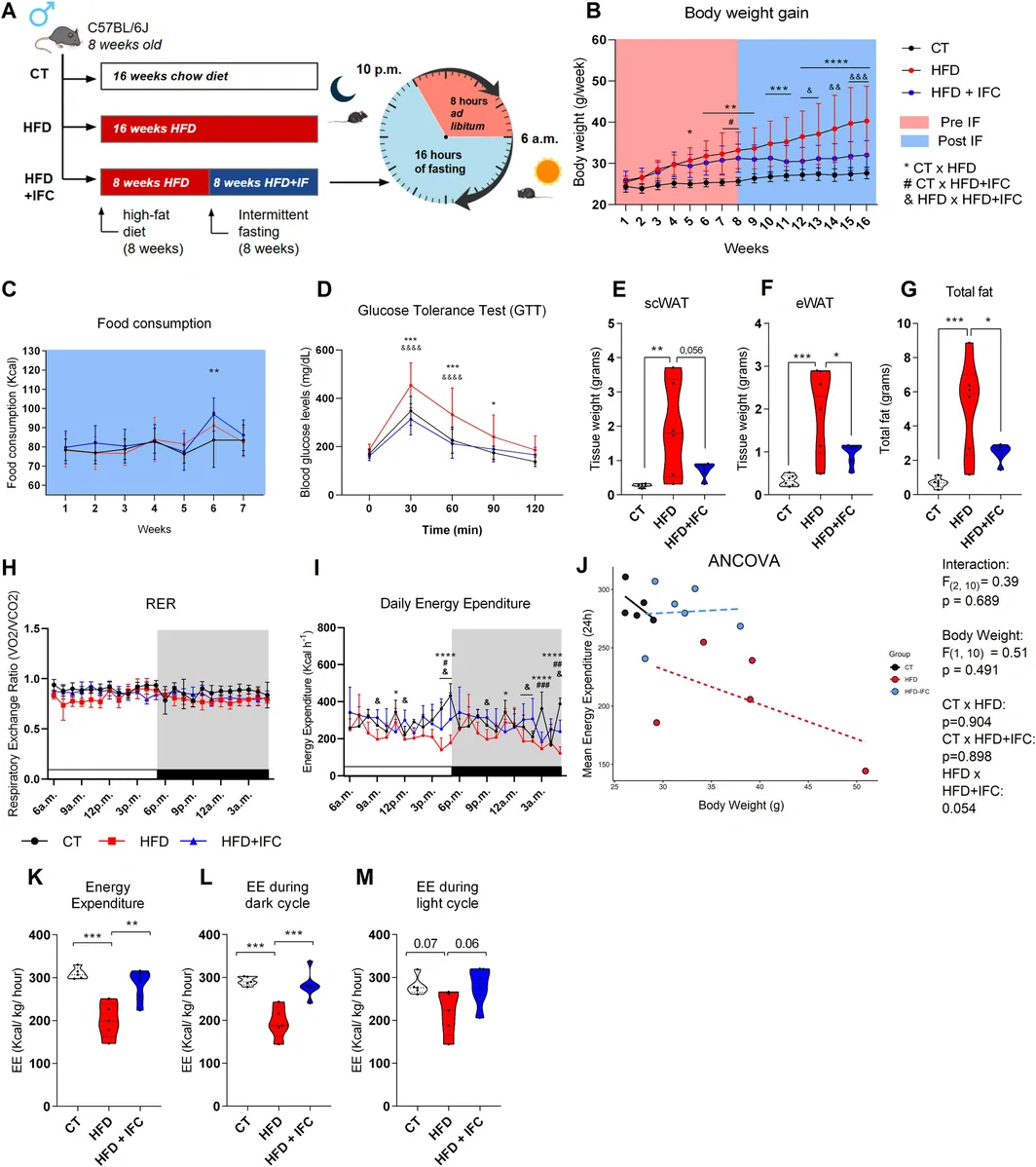
Physiological parameters after chronic intermittent fasting protocol. (A) Experimental design of C57BL/6J male mice under 16 weeks of chow diet (CT), 16 weeks of high‐fat diet (HFD), or 8 weeks of high‐fat diet followed by 8 weeks of high‐fat diet under 16:8 daily intermittent fasting protocol; (B) Body weight gain during 16 weeks (*N* = 15); (C) Food consumption under 7 weeks of intermittent fasting protocol (*N* = 15); (D) Glucose tolerance test (GTT) (*N* = 10); (E) Subcutaneous adipose tissue (scWAT) weight (*N* = 5 and 6); (F) Epididymal adipose tissue (eWAT) weight (*N* = 5 and 6); (G) Total body fat weight (*N* = 5 and 6); (H) 24 h respiratory exchange ratio (RER) analysis (*N* = 5 and 6); (I) 24 h energy expenditure (EE) (*N* = 5 and 6); (J) ANCOVA Type III analysis for energy expenditure and body weight (*N* = 16); (K) Energy expenditure (*N* = 5 and 6); (L) Energy expenditure during dark cycle (*N* = 5 and 6); (M) Energy expenditure during light cycle (*N* = 5 and 6). Differences between groups are indicated as follows: * for CT versus HFD; # CT versus HFD + IFC; & HFD versus HFD + IFC; Statistical significance: *p < 0.05, **p < 0.01, ***p < 0.001, and ****p < 0.0001.

The HFD increased the weight of epididymal white adipose tissue (eWAT) and subcutaneous white adipose tissue (scWAT) compared with controls (Figure 1E,F). However, intermittent fasting prevented the increase in eWAT, as well as total adipose tissue mass (Figure 1E–G). Although the groups did not differ in the 24‐h respiratory exchange ratio (RER) (Figure 1H), HFD‐fed mice exhibited transient reductions in energy expenditure at specific time points during the dark phase compared with control animals, whereas no differences were observed during the light phase (Figure 1I).

Next, we employed a Type III ANCOVA analysis using body weight as a covariate (Figure 1J). After adjustment for body weight, there was no statistically significant effect of the experimental groups on energy expenditure (*F*(_2,12_) = 0.39, *p* = 0.689, pairwise comparisons with *p* > 0.05). This result indicates that the shifts in energy expenditure could be tightly coupled with the changes in body mass induced by the protocols. However, a pairwise comparison between the HFD and HFD‐IFC groups revealed a marginal trend toward increased EE in the fasted group (*p* = 0.054) (Figure 1J). Notably, while the HFD group led to a clear descriptive depression in adjusted metabolic output relative to its larger body mass, the HFD‐IFC group effectively countered this inefficiency, maintaining estimated marginal energy expenditure stable, and close to control levels. After normalization to total body weight, HFD significantly reduced mean daily energy expenditure compared with the control group, whereas intermittent fasting increased energy expenditure relative to HFD‐fed mice (Figure 1K). A similar pattern was observed during the dark phase (Figure 1L). In contrast, no significant differences were detected among groups for mean energy expenditure during the light phase after normalization to body weight (Figure 1M). Together, the analysis suggests that the intermittent fasting protocol may have effects on energy expenditure that are not fully explained by changes in body weight; however, the absence of a significant effect after adjustment for body weight does not provide evidence for a weight‐independent therapeutic effect.

### 3.2 Intermittent Fasting Regulates Thermogenic and Mitochondrial Dynamic Markers in BAT

The HFD‐fed mice (both HFD and HFD‐IFC groups) exhibited a significant increase in body temperature (Figure 2A), while only the HFD group showed an increased BAT mass after 8 weeks of intervention (Figure 2B). Although BAT mass was not significantly reduced in the IFC group (Figure 2B), molecular alterations were observed. There were no observed differences in mRNA levels of *Ucp1* (Figure 2C), carnitine palmitoyltransferase I alpha (*Cpt1α*) (Figure 2D), or peroxisome proliferator‐Activated receptor Gamma coactivator 1‐Alpha (*Ppargc1a*) (Figure 2E) at gene expression levels in BAT. The content of UCP1 and CPT1α proteins did not differ significantly in the BAT of obese animals compared with the control group. However, the intermittent fasting increased the protein content of UCP1 in the BAT, in comparison with the HFD group (Figure 2F,G). In addition, intermittent fasting increased CPT1α protein content in BAT, when compared to the control group (Figure 2F,H). Furthermore, no differences in PGC1α protein content were detected in the BAT of the groups studied (Figure 2I).

**FIGURE 2:**
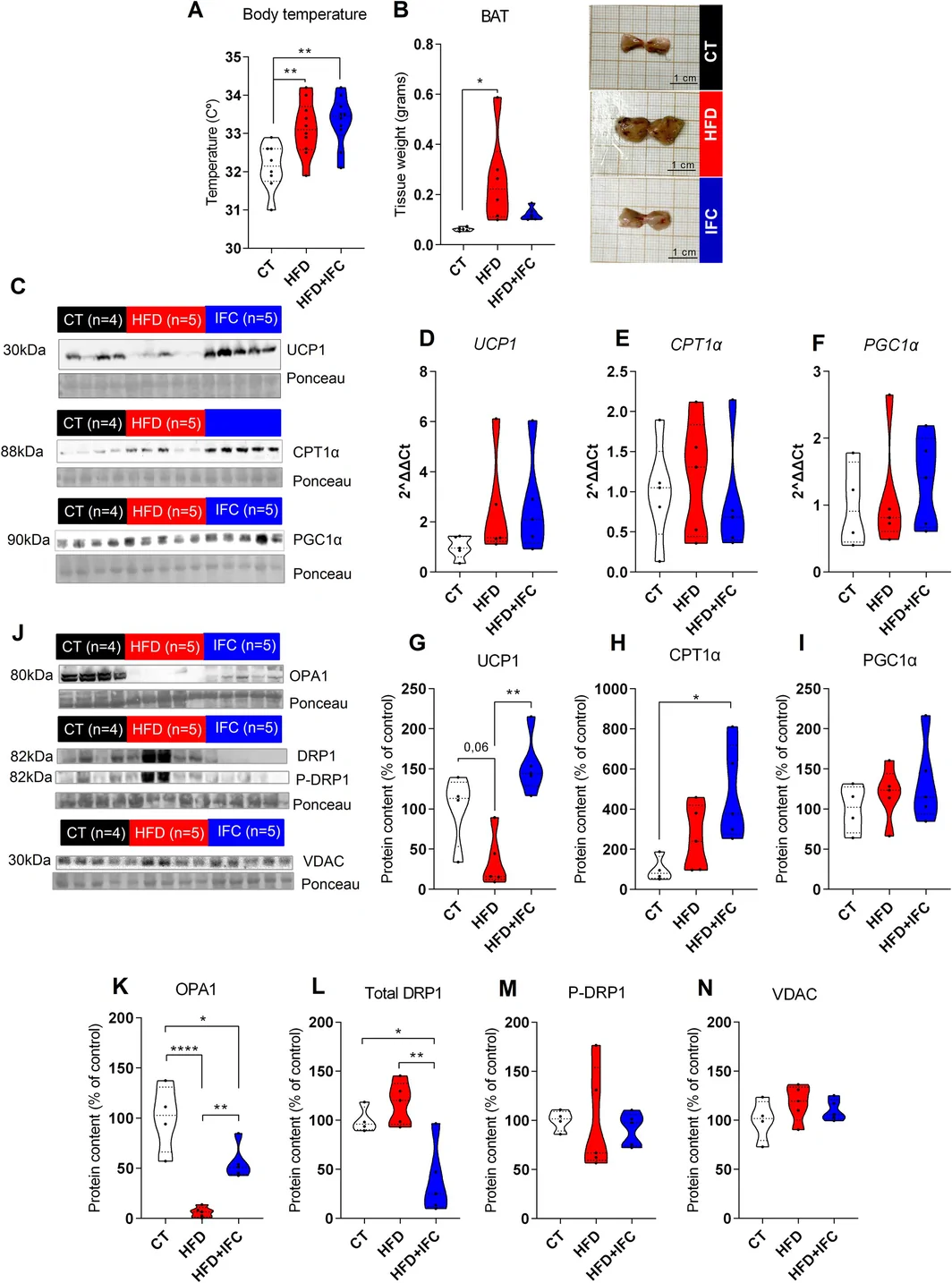
Mitochondrial thermogenic and mitochondrial dynamics gene and protein markers of brown adipose tissue in mice in chow, high‐fat diet, or high‐fat diet during 16:8 fasting protocol. (A) Body temperature from the dorsal region (*N* = 8); (B) Brown adipose tissue (BAT) weight (*N* = 5 and 6); (C) Gene expression by RT‐qPCR of UCP1 (*N* = 5); (D) Gene expression by RT‐qPCR of CPT1α (*N* = 5); (E) Gene expression by RT‐qPCR of PGC1α (*N* = 4 and 5); (F) Western blotting analysis of UCP1, CPT1α, and PGC1α protein content (*N* = 4 and 5); (G) Western blotting analysis of UCP1 protein (*N* = 4 and 5); (H) Western blotting analysis of CPT1α protein (*N* = 4 and 5); (I) Western blotting analysis of PGC1α protein (*N* = 4 and 5); (J) Western blotting analysis of OPA1, total DRP1, DRP1 phosphorylated (P‐DRP1), and VDAC protein content (*N* = 4 and 5); (K) Western blotting analysis of OPA1 protein (*N* = 4 and 5); (L) Western blotting analysis of total DRP1 protein (*N* = 4 and 5); (M) Western blotting analysis of P‐DRP1 protein (*N* = 4 and 5); (N) Western blotting analysis of VDAC protein (*N* = 4 and 5); Statistical significance: *p < 0.05, **p < 0.01, ***p < 0.001, and ****p < 0.0001.

To further investigate the mitochondrial remodeling, we characterized classic markers of mitochondrial dynamics (Figure 2J). The protein content of OPA1, a key mediator of mitochondrial fusion, was significantly decreased by the HFD but was partially rescued in the HFD‐IFC group (Figure 2K). Meanwhile, the HFD‐IFC group showed a significant reduction in total DRP1 levels compared to both the CT and HFD groups (Figure 2L), while phosphorylated DRP1 (P‐DRP1) remained unchanged (Figure 2M). Lastly, to evaluate if these dynamic alterations were linked to changes in mitochondrial quantity rather than quality, we monitored VDAC protein levels in the BAT, which showed no differences between the groups (Figure 2N). Collectively, these data demonstrated that intermittent fasting preserves BAT mitochondrial homeostasis by regulating mitochondrial dynamics protein markers through OPA1, while reducing the expression of the fission protein DRP1, alongside promoting consistent alteration in UCP1 and CPT1α protein content in BAT, suggesting the activation of thermogenesis UCP1‐mediated and fatty‐acid oxidation to prevent diet‐induced oxidative damage [47, 48].

### 3.3 Obesity Increases Lipid Accumulation in BAT, Whereas Intermittent Fasting Promotes Adipose Remodeling by Restoring Physiological Lipid Handling

Next, a histological study of the BAT was performed using H&E staining. Through visual inspection, it is possible to observe an increase in white fat deposition in the BAT of obese animals, while the fasting protocol partially reversed this process (Figure 3A). A more detailed analysis confirmed that obesity increased the mean lipid droplet size in BAT (Figure 3B) and reduced the proportion of smaller lipid droplets (< 30 μm^2^) compared with the CT and HFD+IFC groups (Figure 3C). Notably, intermittent fasting promoted adipose remodeling in BAT, restoring lipid droplet size and reducing the proportion of lipid droplets > 30 μm^2^ (Figure 3D–F).

**FIGURE 3:**
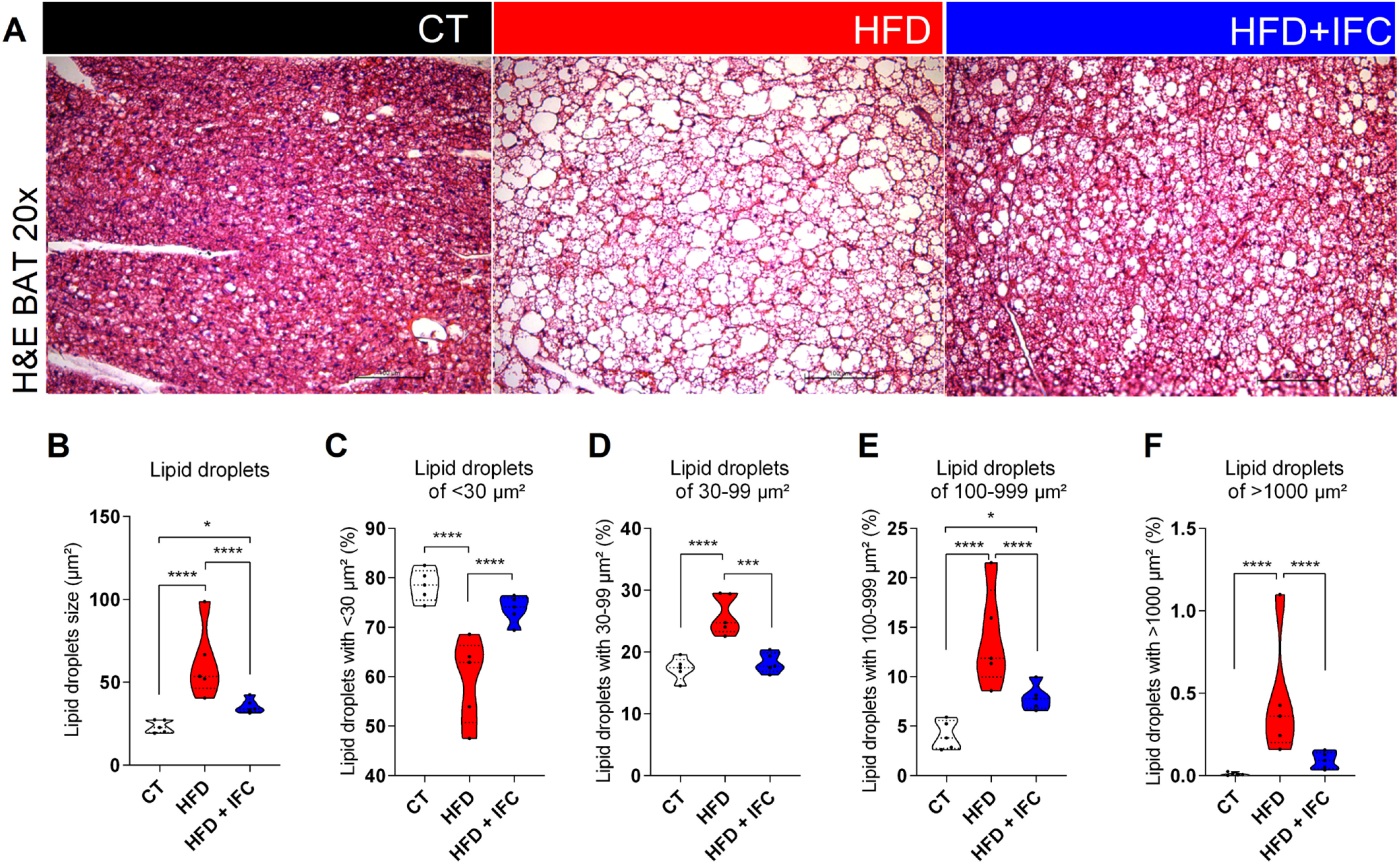
Hematoxylin & eosin analysis and lipid droplets quantification of mice in chow, high‐fat diet, or high‐fat diet during 16:8 fasting protocol. (A) H&E representative images of brown adipose tissue (BAT) from C57BL/6J male mice under 16 weeks of chow diet (CT), 16 weeks of high‐fat diet (HFD) or 8 weeks of high‐fat diet followed by 8 weeks of high‐fat diet under 16:8 daily intermittent fasting under 20× zoom (1 mm); (B) Total lipid droplets size content in μm^2^ (*N* = 5); (C) Percentage of small lipid droplets content (< 30μm^2^) (*N* = 5); (D) Percentage of medium lipid droplets content (30–99 μm^2^) (*N* = 5); (E) Percentage of large lipid droplets content (100–999 μm^2^) (*N* = 5); (F) Percentage of extra‐large lipid droplets content (> 1000μm^2^) (*N* = 5). Statistical significance: *p < 0.05, **p < 0.01, ***p < 0.001, and ****p < 0.0001.

### 3.4 Intermittent Fasting Promotes Mitochondrial Morphology Remodeling and Improves Cristae Organization

To evaluate the effects of intermittent fasting on mitochondrial morphological modulation, the transmission electron microscopy (TEM) analysis was performed (Figure 4). An altered mitochondrial morphology was observed in the HFD group compared to the control group, which was recovered by the intermittent fasting (Figure 4A). Despite no differences regarding mitochondrial size (μm^2^) between groups (Figure 4B), an increase in the mitochondrial aspect ratio was observed in the HFD+IFC group compared to HFD, with a higher ratio indicating a more elongated mitochondrial format after the intermittent fasting, even without differences in the form factor (Figure 4C,D). Accordingly, the mitochondrial circularity ratio was increased in the HFD group compared to the control animals; however, the circularity ratio profile in the intermittent fasting group was similar to both groups (Figure 4E). While HFD reduced the mitochondrial elongation ratio when compared to the control group, the intermittent fasting protocol reversed the elongation and Feret's diameter compared to HFD, with mitochondrial features similar to the CT group (Figure 4F,G).

**FIGURE 4:**
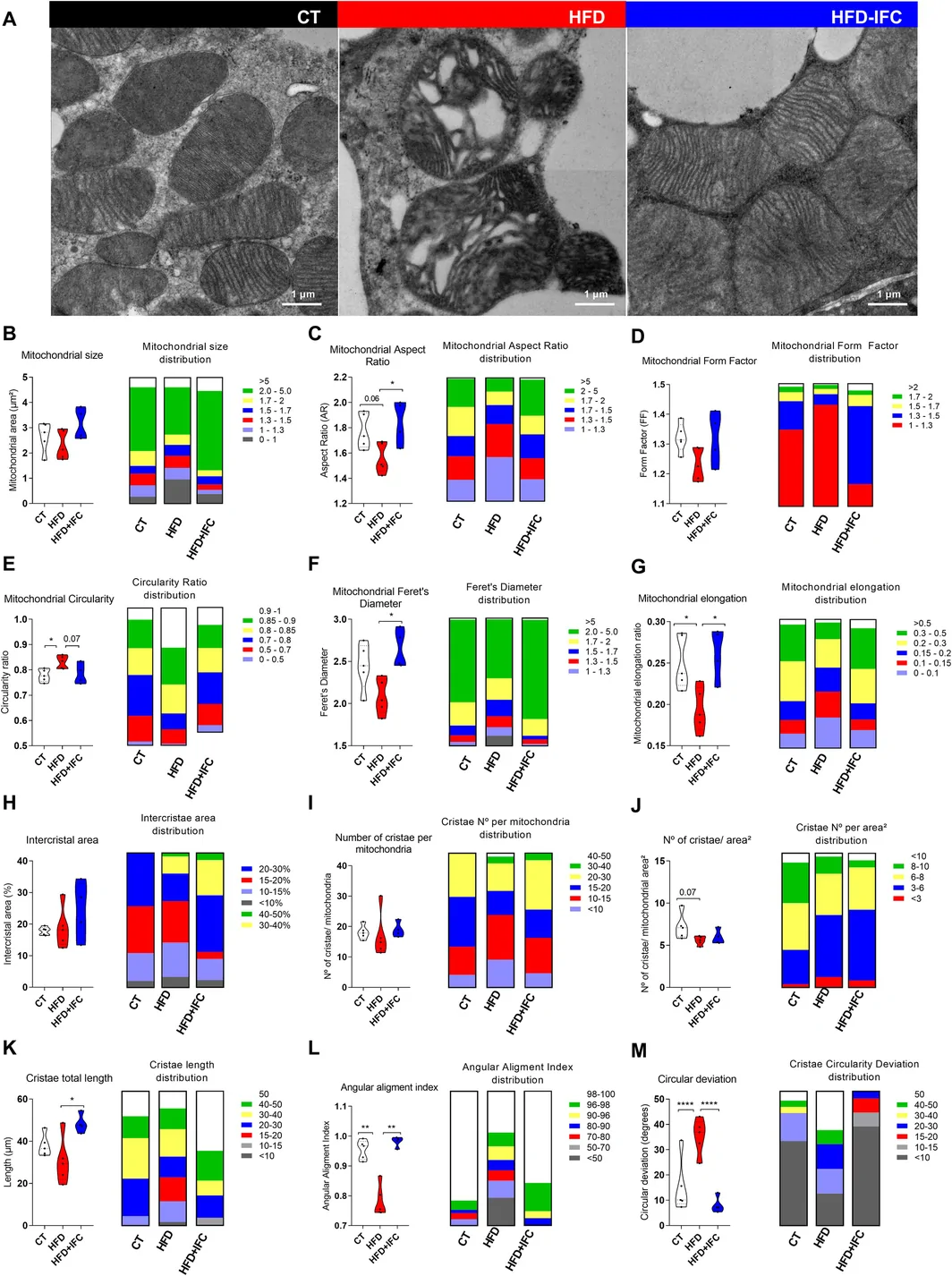
Transmission electron microscopy (TEM) and mitochondrial structure and cristae parameters quantification of mice in chow, high‐fat diet, or high‐fat diet during 16:8 fasting protocol. (A) Transmission electron microscopy (TEM) representative images of brown adipose tissue (BAT) from C57BL/6J male mice under 16 weeks of chow diet (CT), 16 weeks of high‐fat diet (HFD) or 8 weeks of high‐fat diet followed by 8 weeks of high‐fat diet under 16:8 daily intermittent fasting (*N* = 4 and 5); (B) Mitochondrial size quantification and distribution; (C) Mitochondrial aspect ratio quantification and distribution; (D) Mitochondrial form factor quantification and distribution; (E) Mitochondrial circularity quantification and distribution; (F) Mitochondrial Feret's diameter quantification and distribution; (G) Mitochondrial elongation quantification and distribution; (H) Intercristae area quantification and distribution; (I) Number of cristae per mitochondria quantification and distribution; (J) Number of cristae per mitochondrial size quantification and distribution; (K) Cristae total length quantification and distribution; (L) Cristae Angular Alignment Index quantification and distribution; (M) Cristae circular deviation quantification and distribution. Statistical significance: *p < 0.05, **p < 0.01, ***p < 0.001, and ****p < 0.0001.

Next, we evaluated the mitochondrial cristae aspects in the BAT. In our analysis, no differences were found in terms of intercristae area or the number of cristae per mitochondria or per area among the experimental groups (Figure 4H–J). However, intermittent fasting increased the cristae length when compared to the HFD animals (Figure 4K). Moreover, obesity markedly disrupted cristae alignment and angular distribution, as evidenced by reduced cristae angular alignment and increased cristae circular deviation in the HFD group compared with the control group, indicating disorganization of mitochondrial cristae structure (Figure 4L,M). In contrast, intermittent fasting promoted cristae remodeling, increasing cristae alignment and reducing circular deviation, suggesting substantial recovery of mitochondrial cristae organization, along with improvements in mitochondrial structure and function.

### 3.5 Intermittent Fasting Increases the Maximum Intrinsic Capacity of Respiratory Complexes in Brown Adipose Tissue and Reduces Oxidative Stress Through Lipid Peroxidation

To evaluate the effects of intermittent fasting on the maximum capacity of individual enzymatic mitochondrial complexes in brown adipose tissue, the high‐resolution respirometry analysis was performed (Figure 5A). The analysis showed an expressive increase of respiratory capacity in the HFD+IFC group compared with both CT and HFD groups, with a higher oxygen consumption by mitochondrial complexes I and II with the addition of the substrate succinate, as well as the complex II isolation by the addition of the complex I inhibitor rotenone (Figure 5B). Furthermore, the addition of octanoylcarnitine (OCT) revealed an enhanced capacity for mitochondrial fatty acid oxidation downstream of carnitine transport in the IFC group (Figure 5B).

**FIGURE 5:**
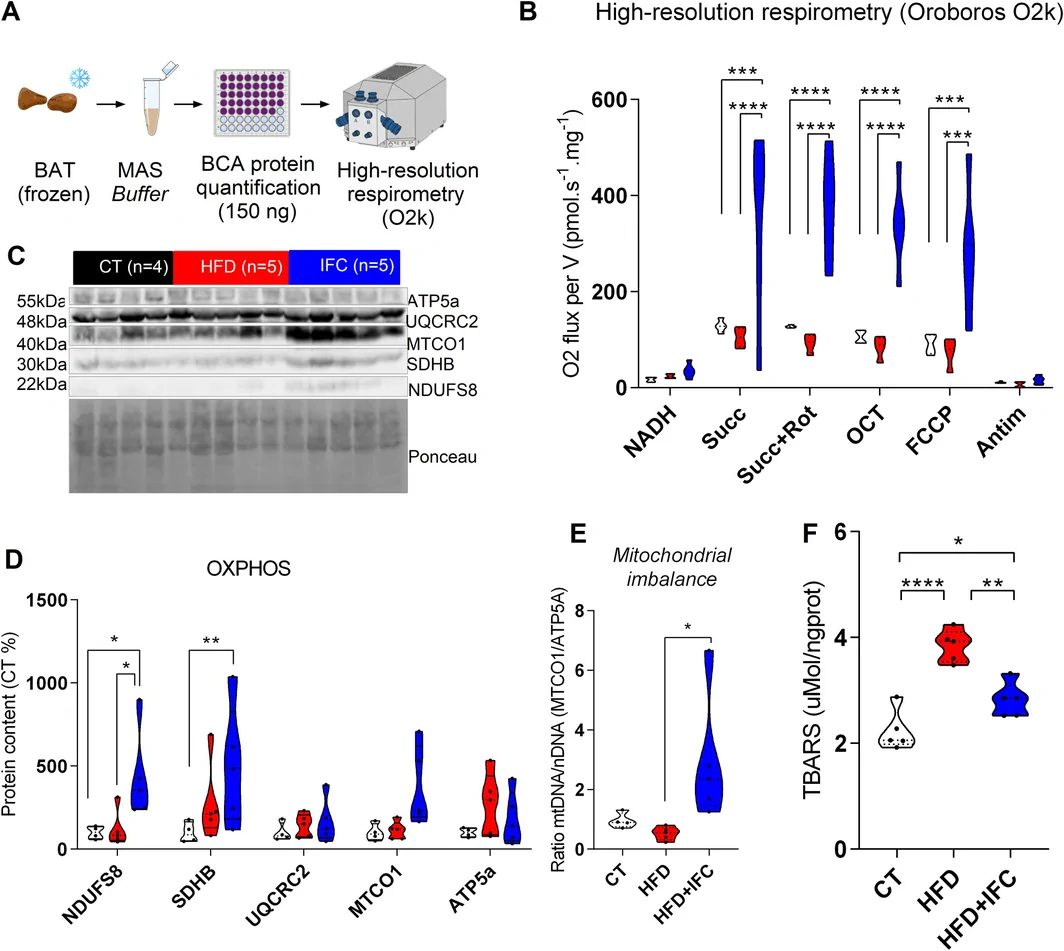
High‐resolution respirometry (Oroboros O2k), mitonuclear protein imbalance quantification, PCA analysis, and OXPHOS protein content of mice in chow, high‐fat diet, or high‐fat diet during 16:8 fasting protocol. (A) Experimental design of frozen brown adipose tissue (BAT) from C57BL/6J male mice under 16 weeks of chow diet (CT), 16 weeks of high‐fat diet (HFD) or 8 weeks of high‐fat diet followed by 8 weeks of high‐fat diet under 16:8 daily intermittent fasting, followed by preparation in MAS buffer, bicinchoninic acid (BCA) protein assay for quantification and high‐resolution respirometry analysis; (B) High‐resolution respirometry of complex I (NADH), complex I+II (Succinate), complex II (Succinate+Rotenone), fatty‐acid oxidation (OCT), maximal respiratory capacity (FCC) and non‐mitochondrial respiration (Antimycin) (*N* = 6); (C) Western blotting of OXPHOS protein content (*N* = 4 and 5); (D) Western blotting analysis of OXPHOS protein content (*N* = 4 and 5); (E) Mitonuclear protein imbalance using mtDNA/nDNA ratio (*N* = 6); (F) Thiobarbituric acid reactive substances (TBARS) assay (*N* = 5). Statistical significance: *p < 0.05, **p < 0.01, ***p < 0.001, and ****p < 0.0001.

Consistent with the individual mitochondrial complexes respiratory analysis, the mitochondrial electron transport chain (ETC) protein levels were increased in BAT from the HFD + IFC group, with an upregulation of complex I subunit NDUFS8 compared to CT and HFD, and the complex II subunit SDHB compared to the CT group (Figure 5C,D). Taken together, these data demonstrate that fasting increased proteostasis of the OXPHOS‐related proteins and the respiratory capacity of mitochondrial complexes in BAT.

Considering the positive alteration in the proteostasis of oxidative phosphorylation components (OXPHOS), coupled with the morphological adaptations of mitochondria in response to fasting, we suspect that intermittent fasting could induce the mitonuclear protein imbalance in BAT. Mitonuclear protein imbalance is an adaptive process to mitochondrial stress, a highly conserved process across species, which is generally detected by an imbalance in the stoichiometric ratios of OXPHOS‐related proteins encoded by nuclear DNA (nDNA) and mitochondrial DNA (mtDNA) [49, 50]. Thus, the stoichiometric ratio between mitochondrial DNA–encoded and nuclear DNA–encoded proteins (mtDNA/nDNA) was determined in the BAT samples. This analysis revealed that intermittent fasting elicited the mitonuclear protein imbalance in BAT, compared to the HFD group (Figure 5E). To further investigate whether this adaptive mitonuclear protein imbalance and enhanced respiratory capacity were accompanied by oxidative stress, we evaluated the lipid peroxidation in BAT using the TBARS assay (Figure 5F). This analysis revealed that HFD treatment increased the oxidative stress in the BAT, conversely chronic intermittent fasting reduced the oxidative stress in the BAT (Figure 5F). Collectively, these findings demonstrated that chronic intermittent fasting improves mitochondrial proteostasis by modulating OXPHOS components, increased the mitochondrial respiratory capacity and reduced oxidative damage in BAT.

## 4 Discussion

Brown adipose tissue plays a key role in energy expenditure and weight loss, with multiple studies demonstrating its potential to modulate healthy body homeostasis during metabolic diseases, such as obesity. The mitochondrial dynamics and metabolism in BAT are crucial not only for the control of thermogenesis, but its regulation is essential to prevent a defective respiratory capacity, increased ROS production, and impaired mitochondrial functionality [51, 52, 53]. Accordingly, our study investigated the potential effects of chronic intermittent fasting (IFC) in the brown adipose tissue of obese mice. Our data revealed that the intermittent fasting protocol not only restores BAT lipid composition but also promotes mitochondrial reprogramming. While HFD disturbed the morphofunctional parameters of different mitochondria, intermittent fasting improved electron transport chain (ETC) function, stimulated thermogenic protein expression, and preserved the mitochondrial ultrastructure.

As expected, obesity increased the lipid droplet accumulation in BAT. It was accompanied by smaller, fragmented, and abnormal mitochondrial structures. Previous studies have shown that intermittent fasting improves mitochondrial respiratory capacity in adipose tissues, including increased oxygen consumption in beige adipocytes from obese mice [28] and enhanced respiratory activity in subcutaneous adipose tissue [54]. The increased mitochondrial respiration observed in the intermittent fasting group suggests enhanced sympathetic activation, likely stimulating fatty acid oxidation, consistent with the reduced lipid accumulation observed. Considering the association of mitochondrial fusion to promote fatty acid oxidation during starvation [55], it is expected that the magnitude of the respiratory increase during intermittent fasting is associated with fatty acid oxidation promoted by mitochondrial remodeling. In this context, intermittent fasting may restore mitochondrial network integrity, thereby enhancing lipid oxidation and thermogenic competence, promoting an increase in mitochondrial respiratory capacity. Moreover, studies have shown that intermittent fasting can induce spontaneous caloric restriction. Although reduced food intake was not observed in the present study, the metabolic switch from glucose to fatty acids and ketone bodies during intermittent fasting has been proposed to modulate mitochondrial function and bioenergetics [56].

A previous study showed that mitochondria in epididymal WAT from *ob*/*ob* mice displayed a similar phenotype, with dysregulated mitochondrial dynamics marked by excessive fission and fragmentation, as evidenced by the accumulation of rounded mitochondria [57]. Comparable alterations have also been reported in other adipose depots. In perivascular WAT, evidence of downregulation of UCP1 protein content due to a high‐fructose diet in mice was intrinsically related to the upregulation of mitochondrial fission proteins [58]. The excessive fragmentation through RalA (a GTPase involved in cell signaling) activation also leads to mitochondrial dysfunction in inguinal WAT of obese mice, contributing to weight gain [52]. Impaired visceral WAT homeostasis caused by the loss of TIPE2, a negative regulator of immune responses, in macrophages also increased mitochondrial fission, leading to elevated ROS production, and ferroptosis [59]. Collectively, these studies reinforce that excessive mitochondrial fission is a common feature of adipose tissue dysfunction during obesity.

Another study reported beneficial effects from partially controlling mitochondrial damage induced by excessive fission, suggesting that strategies aimed at restoring the balance between fusion and fission may be critical for maintaining mitochondrial energy homeostasis, as this approach was shown to ameliorate neurodegeneration [60]. Although excessive fission is detrimental to mitochondrial energetic balance, the absence of the fission process is also dangerous for mitochondrial health, as it was observed in Drp1‐KO mice, highlighting the importance of mitochondrial dynamics balance [61]. Considering these findings and according to this study, obesity can compromise mitochondrial metabolism in several adipose tissues by promoting mitochondrial dynamics modulation, which can indicate a potential mitochondrial fission and lead to a disrupted balance in mitochondrial dynamics.

Furthermore, a previous study showed that in UCP1 KO mice, there was not only a clear aberrant cristae morphology in BAT, but also impaired NADH and ATP synthesis pathways [62]. As shown by our data, obesity was associated with a similar morphological pattern, characterized by fragmented mitochondria and disorganized cristae. Interestingly, intermittent fasting in obese mice showed a completely different mitochondrial morphofunctional profile, with the upregulation of UCP1 protein content, an expressive increase of ETC respiratory capacity, and complex I and II protein contents [63]. Also, mitochondria displayed elongated and organized cristae. Several proteins coordinate mitochondrial cristae organization, where MICOS (Mitochondrial Contact Site and Cristae Organizing System), a set of proteins that integrate the mitochondrial intermembrane space, plays the central role in cristae remodeling [62]. The protein MICU1, part of MICOS and responsible for regulating cristae organization in a Ca^2+^−dependent manner, is assisted by UCP2 through an excessive Ca^2+^ release and opening of cristae junctions through MICU1 methylation [64]. Although there is no clear evidence that UCP1 directly interacts with MICOS or MCU proteins, UCP1 has been linked to key regulators of MCU, such as the UCP1–EMRE–MCU complex, suggesting a possible role for UCP1 in cristae remodeling [63, 65]. The excessive lipid deposition during obesity can also be a strong factor contributing to cristae remodeling through MICOS, with a shared lipid‐dependent pathway regulating both UCP1 expression and activity and cristae dynamics [64, 66], which could be explored further in future studies.

Prolonged overnutrition during obesity also appears to drive time‐dependent remodeling of thermogenic regulation. Whereas a previous study demonstrated that 4 weeks of high‐fat feeding induces a compensatory increase in UCP1‐mediated thermogenesis during the early stages of obesity [24], our results indicate that 16 weeks of diet‐induced obesity tended to reduce UCP1 protein abundance (*p* = 0.06). These findings suggest that BAT thermogenesis undergoes dynamic remodeling during obesity progression, with an initial activation of canonical UCP1‐dependent thermogenesis followed by progressive BAT dysfunction characterized by whitening, chronic inflammation, and impaired non‐shivering thermogenesis [67].

It is important to highlight that, although UCP1 in BAT has been considered a key protein in the control of thermogenesis, recent studies suggest the existence of alternative mechanisms regulating adaptive thermogenesis, since UCP1‐deficient mice were able to generate heat [68]. In addition, it has been proposed that creatine metabolism in beige and BAT is capable of generating heat by consuming ATP in a futile cycle [69, 70, 71]. This mechanism is coordinated by the phosphatase TNAP, a critical enzyme responsible for the phosphocreatine hydrolysis [72]. Furthermore, Liu and colleagues reported that peroxisomal metabolism of branched‐chain fatty acids also contributes to adaptive thermogenesis and systemic energy homeostasis independently of UCP1 [73]. These data illustrate the complexity of BAT in the control of thermogenesis.

Interestingly, in this study we observed that intermittent fasting significantly increased protein levels of UCP1 and CPT1a, which may reflect a temporal dissociation between rapid molecular adaptation and slower morphological remodeling [68, 74]. The elevated expression of UCP1 and CPT1a in the HFD‐IFC group suggests that while the tissue is molecularly primed and actively executing lipid oxidation, a longer intervention period might be required to fully translate these enzymatic changes into macroscopically visible tissue remodeling. Thus, these findings suggest a transient UCP1 regulation in BAT during obesity, within an induction as an initial compensatory activation of thermogenesis, followed by adaptive downregulation, through UCP1‐independent thermogenesis regulation [71, 75, 76, 77, 78]. Considering that, future studies should characterize the temporal dynamics of thermogenic regulation during obesity progression.

Given the importance of maintaining the plasticity of mitochondrial dynamics for mitochondrial function and regulation of thermogenic metabolism, strategies to ameliorate mitochondrial dysfunction are crucial to mitigating the detrimental effects of obesity. A previous study showed that nicotinamide riboside (NR) supplementation can improve both NAD^+^ metabolism and mitochondrial function in BAT [24]. Intermittent fasting has also been shown to improve mitochondrial function by enhancing fusion processes through Sirt3 in inguinal WAT [17], increasing UCP1 content in subcutaneous WAT, reducing inflammation, improving insulin sensitivity [18], and enhancing postprandial thermogenesis in BAT [12].

Our data demonstrated that intermittent fasting promoted profound mitochondrial remodeling, alterations in mitochondrial proteostasis, and increased the maximum intrinsic capacity of individual respiratory complexes in the BAT of obese mice. Remarkably, these adaptations resemble the mitochondrial remodeling observed following induction of mitonuclear protein imbalance. For example, inducers of mitonuclear protein imbalance, such as doxycycline, nicotinamide riboside (NR) [49, 50], and physical exercise [79, 80, 81], alter the stoichiometry of OXPHOS proteins, thereby triggering adaptive mitochondrial stress responses. To our knowledge, the present study is the first to demonstrate that intermittent fasting induces a mitonuclear protein imbalance signature in BAT. Although our findings establish an association between intermittent fasting and mitonuclear protein imbalance, whether this mechanism directly mediates the improvements in mitochondrial function or represents one component of a broader adaptive mitohormetic response remains to be determined.

## 5 Study Limitations

The use of frozen tissues in mitochondrial respirometry analyses is a limiting factor of the study. Although freezing can compromise mitochondrial membrane integrity and the organization of respiratory supercomplexes, frozen samples can provide important and reliable information regarding the individual capacity of the electron transport chain [44, 45, 46], as demonstrated in this study.

Moreover, while our molecular, structural, and mitochondrial dynamics markers (associated with fission and fusion processes) data point toward metabolic improvements, functional in vivo assessments of BAT activation, such as cold‐exposure challenges, were not performed, which represents an important direction for future physiological validation.

Another important point is that in the energy expenditure (EE) assessment experiment, we observed an atypical EE pattern during the dark phase. Healthy animals typically exhibit an increase in EE, particularly during the first few hours of the dark period. However, in our experiment, the control group did not show a consistent increase in EE during the dark cycle; we observed only sporadic increases during the first and last time points of the dark cycle. Finally, while our study focused specifically on the structural and mitochondrial remodeling of BAT, we did not characterize other adipose depots, such as white adipose tissue (WAT). Given the substantial contribution of WAT to systemic glucose and lipid metabolism, the lack of WAT depot weights, histology, and metabolic markers represents a limitation. Future studies investigating WAT dynamics are warranted to determine whether the observed metabolic benefits of intermittent fasting are driven primarily by BAT‐specific changes or represent a coordinated response across multiple adipose depots.

## 6 Conclusion

Obesity markedly disrupted mitochondrial architecture in BAT, leading to smaller, more spherical organelles with disorganized cristae. In contrast, intermittent fasting reduced BAT adiposity while regulating mitochondrial thermogenic and dynamic‐related protein levels and restoring a brown‐like phenotype. Notably, intermittent fasting induced profound remodeling of mitochondrial dynamics, characterized by enhanced mitochondrial elongation, increased total cristae length, and improved cristae organization. Furthermore, intermittent fasting augmented the maximal intrinsic respiratory capacity of mitochondrial complexes and increased the abundance of oxidative phosphorylation–related proteins. Collectively, these findings support intermittent fasting as a promising strategy to mitigate mitochondrial dysfunction in BAT under metabolic stress conditions.

## Author Contributions

**Fernanda Silva Carneiro:** conceptualization, methodology, data curation, investigation, validation, formal analysis, visualization, writing – original draft, writing – review and editing. **Larissa Moreira Dias:** conceptualization, methodology, data curation, formal analysis, investigation, visualization, writing – original draft, writing – review and editing. **Scylas José de Andrade Junior:** methodology, investigation, data curation, writing – review and editing. **Matheus Biscaro Rocha:** methodology, investigation, data curation, writing – review and editing. **Mariana Lima Ribeiro:** methodology, data curation, investigation, writing – review and editing. **Ana Paula Morelli:** methodology, data curation, investigation, writing – review and editing. **Gabriel Calheiros Antunes:** methodology, investigation, data curation, writing – review and editing. **Adelino Sanchez Ramos Da Silva:** formal analysis, data curation, writing – review and editing. **Dennys Esper Cintra:** formal analysis, data curation, supervision, writing – review and editing. **José Rodrigo Pauli:** formal analysis, data curation, visualization, methodology, funding acquisition, resources, supervision, writing – review and editing. **Eduardo Rochete Ropelle:** conceptualization, methodology, data curation, formal analysis, validation, project administration, visualization, funding acquisition, supervision, resources, writing – original draft, writing – review and editing.

## Funding

This study was supported by the Sao Paulo Research Foundation (FAPESP) under the grant numbers #2022/02350‐8, #2023/13629‐6, #2024/05542‐0, and #2019/21709‐4, and in collaboration under the grant numbers #2024/21229‐0 and #2023/02751‐5. The work was also supported by the Brazilian National Council for Scientific and Technological Development (CNPq) under the grant number #304123/2023‐4 and the Coordination for the Improvement of Higher Education Personnel (CAPES) under the grant numbers #88887.238630/2025‐00, #88887.142758/2025‐00, and #88887.204283/2025‐00.

## Conflicts of Interest

The authors declare no conflicts of interest.

## Supporting information


**Supplementary File 1.** Detailed methodology for mitochondrial transmission electron microscopy quantification analysis.

**Supplementary File 2.** Representative images of mitochondrial transmission electron microscopy quantification analysis.
**Supplementary Figure 1**. Representative images of transmission electron microscopy (TEM) analysis of control (CT) group (*n* = 5).
**Supplementary Figure 2**. Representative images of transmission electron microscopy (TEM) analysis of high‐fat diet (HFD) group (*n* = 5).
**Supplementary Figure 3**. Representative images of transmission electron microscopy (TEM) analysis of high‐fat diet with intermittent fasting (HFD‐IFC) group (*n* = 4).

**Supplementary Table 1.** Detailed antibodies list for western blotting analysis.
**Supplementary Table 2**. Detailed primers list for RTq‐PCR analysis.

## Data Availability

The data generated and analyzed in this study are available from the corresponding author upon reasonable request.
