# Supplementary material for: Intermittent Fasting Remodels Mitochondrial Morphology and Bioenergetic Capacity in Brown Adipose Tissue of Obese Mice
Source: Acta Physiol (Oxf). 2026 Nov 8;242(12):e70337. doi: 10.1111/apha.70337 (PMC13745782; doi:10.1111/apha.70337)
Supplement: Supplementary file 1 [file APHA-242-e70337-s001.docx]

**Intermittent fasting reprograms mitochondrial dynamics and bioenergetics in brown adipose tissue of obese mice.** Carneiro, FS (2026).

**Supplementary file 1**. Detailed methodology for mitochondrial Transmission Electron Microscopy quantification analysis.

1. **Imaging processing and calibration**

The image analysis was performed using ImageJ/Fiji (version 2.14.0, NIH, USA), with the calibration applied based on the microscope scale bar on every image before all the measurements. The quantification analysis of each parameter was performed according to the following data. All measurements were performed in a blinded manner, with approximately 200 mitochondria for each sample for morphological analysis, and 50 mitochondria resulting in 350-800 individual cristae analyzed per group for detailed cristae analysis.

2. **Mitochondrial morphological analysis**

**2.1. Mitochondrial area**

The mitochondrial area was segmented using a semi-automated method, combined the use of the plug-in Skeletonized (2D/3D) with manual refinement, using the polygon selection tool and calculated automatically by the software.

**2.2. Aspect ratio**

The aspect ratio was calculated between the major and minor axis, both automatically analyzed by the software.

*Aspect ratio = major axis / minor axis*

**2.3. Form factor**

The form factor was calculated as (perimeter²) / (4π × area), based on the geometric relation between the perimeter and the area, in which a perfect circle yields a value of 1, and the deviations increasing the value reflecting a more irregular and complex form.

*Form factor = (Perimeter²) / (4π × Area)*

**2.4. Circularity ratio**

The circularity ratio was calculated as 4π × (Area / Perimeter²), indicating circular mitochondria with values closer to 1.

*Circularity = 4π × (Area / Perimeter²)*

**2.5. Feret’s diameter**

The Feret’s diameter is automatically analyzed by the software, defined by the maximum distance between the two parallel tangents on the opposite sides of the mitochondria.

**2.6. Elongation index**

The elongation index is calculated by 1 − (minor axis / major axis), indicating the elongation based on the relation between the two axes of the ellipse.

*Elongation = 1 − (minor axis / major axis)*

3. **Cristae ultrastructure and organization analysis**

**3.1. Intercristae area**

The intercristae area is measured as the distance between adjacent cristae from a mitochondria, with the regions calculated combined the use of the plug-in Skeletonized (2D/3D) with manual refinement, automatically calculated by the software.

**3.2. Total cristae number**

The total number of cristae of each mitochondria were counted automatically by the software.

**3.3. Cristae number per mitochondrial area**

The cristae number per mitochondrial area was calculated as the relation between the total number and the area of each mitochondria.

*Cristae density = total cristae number / mitochondrial area*

**3.4. Total cristae length**

The total cristae length of each mitochondria was counted automatically by the software.

**3.5. Angular Alignment Index**

The angular alignment index of mitochondria cristae was calculated as the mean deviation of cristae angles relative to the mitochondrial longitudinal axis, with lower values indicating higher alignment.

*Angular Alignment Index = (1 / number of cristae) × Σ |θ crista|*

**3.6. Cristae circular deviation**

Represents the variability in cristae orientation angles within a mitochondrion. It was calculated as the circular standard deviation of the measured angles.

*Cristae circular deviation = √[−2 × ln(R)]*

where *R = √[(mean(cos(θ_crista)))² + (mean(sin(θ_crista)))²]*
