# Supplementary material for: Intermittent Fasting Remodels Mitochondrial Morphology and Bioenergetic Capacity in Brown Adipose Tissue of Obese Mice
Source: Acta Physiol (Oxf). 2026 Nov 8;242(12):e70337. doi: 10.1111/apha.70337 (PMC13745782; doi:10.1111/apha.70337)
Supplement: Supplementary file 2 [file APHA-242-e70337-s002.docx]

**Intermittent fasting reprograms mitochondrial dynamics and bioenergetics in brown adipose tissue of obese mice.** Carneiro, FS (2026).

**Supplementary table 1**. Detailed antibodies list for Western blotting analysis.

| **Antibody** | **Target** | **Host** | **Type** | **MW** | **Dilution** | **Catalog** | **Company** |
| --- | --- | --- | --- | --- | --- | --- | --- |
| UCP1 (D9D6X) | UCP1 | Rabbit | Monoclonal | 30 kDa | 1:1000 | #14670 | Cell Signaling Technology |
| PGC-1α (H-300) | PGC-1α | Rabbit | Polyclonal | 90 kDa | 1:1000 | sc-13067 | Santa Cruz Biotechnology |
| CPT1α (D3B3) | CPT1α | Rabbit | Monoclonal | 88 kDa | 1:1000 | #12252 | Cell Signaling Technology |
| OXPHOS | NDUFB8  SDHB  UQCRC2  MT-CO1  ATP5F1A | Mouse | Monoclonal | 55 kDa  48 kDa  40 kDa  30 kDa  20 kDa | 1:1000 | ab110413 | Abcam |
| OPA1 (D6U6N) | OPA1 | Rabbit | Monoclonal | 80 kDa | 1:1000 | 80471S | Cell Signaling Technology |
| p-DRP1 (S637) | Phosphorylated DRP1 | Rabbit | Monoclonal | 82 kDa | 1:1000 | 4867S | Cell Signaling Technology |
| DRP1 | DRP1 | Rabbit | Monoclonal | 82 kDa | 1:1000 | 8570S | Cell Signaling Technology |
| VDAC | Rabbit | Tabbit | Monoclonal | 30 kDa | 1:1000 | 4866S | Cell Signaling Technology |

**Supplementary table 2**. Detailed primers list for RTq-PCR analysis.

| Primer | Foward (5’ - 3’) | Reverse (3’ - 5’) |
| --- | --- | --- |
| *UCP1* | GCTTTGCCTCACTCAGGATTGG | CCAATGAACACTGCCACACCTC |
| *PPARGC1A (PGC1α)* | GTACTGTCGGTTTCAGAAGTGCC | ATCTCCGCCAACAGCTTCTCCT |
| *CPT1α* | GGCATAAACGCAGAGCATTCCTG | CAGTGTCCATCCTCTGAGTAGC |
