# Supplementary material for: Intermittent Fasting Remodels Mitochondrial Morphology and Bioenergetic Capacity in Brown Adipose Tissue of Obese Mice
Source: Acta Physiol (Oxf). 2026 Nov 8;242(12):e70337. doi: 10.1111/apha.70337 (PMC13745782; doi:10.1111/apha.70337)
Supplement: Supplementary file 3 [file APHA-242-e70337-s003.docx]

**Intermittent fasting reprograms mitochondrial dynamics and bioenergetics in brown adipose tissue of obese mice.** Carneiro, FS (2026).

**Supplementary file 2**. Representative images of mitochondrial Transmission Electron Microscopy quantification analysis.

***Supplementary figure 1****. Representative images of Transmission Electron Microscopy (TEM) analysis of Control (CT) group (n=5).*

***Supplementary figure 2****. Representative images of Transmission Electron Microscopy (TEM) analysis of High-fat diet (HFD) group (n=5).*

***Supplementary figure 3****. Representative images of Transmission Electron Microscopy (TEM) analysis of High-fat diet with intermittent fasting (HFD-IFC) group (n=4).*
